# Identification of ADS024, a newly characterized strain of *Bacillus velezensis* with direct *Clostridiodes difficile* killing and toxin degradation bio-activities

**DOI:** 10.1038/s41598-022-13248-4

**Published:** 2022-06-03

**Authors:** Michelle M. O’Donnell, James W. Hegarty, Brian Healy, Sarah Schulz, Calum J. Walsh, Colin Hill, R. Paul Ross, Mary C. Rea, Ronald Farquhar, Laurent Chesnel

**Affiliations:** 1 APC Microbiome Ireland, University College Cork, Cork, Ireland; 2 Teagasc Food Research Centre, Moorepark, Fermoy, Co. Cork, Ireland; 3 Adiso Therapeutics, Inc., Concord, MA USA

**Keywords:** Drug development, Bacteria

## Abstract

*Clostridioides difficile* infection (CDI) remains a significant health threat worldwide. *C. difficile* is an opportunistic, toxigenic pathogen that takes advantage of a disrupted gut microbiome to grow and produce signs and symptoms ranging from diarrhea to pseudomembranous colitis. Antibiotics used to treat *C. difficile* infection are usually broad spectrum and can further disrupt the commensal gut microbiota, leaving patients susceptible to recurrent *C. difficile* infection. There is a growing need for therapeutic options that can continue to inhibit the outgrowth of *C. difficile* after antibiotic treatment is completed. Treatments that degrade *C. difficile* toxins while having minimal collateral impact on gut bacteria are also needed to prevent recurrence. Therapeutic bacteria capable of producing a range of antimicrobial compounds, proteases, and other bioactive metabolites represent a potentially powerful tool for preventing CDI recurrence following resolution of symptoms. Here, we describe the identification and initial characterization of ADS024 (formerly ART24), a novel therapeutic bacterium that can kill *C. difficile *in vitro with limited impact on other commensal bacteria. In addition to directly killing *C. difficile*, ADS024 also produces proteases capable of degrading *C. difficile* toxins, the drivers of symptoms associated with most cases of CDI. ADS024 is in clinical development for the prevention of CDI recurrence as a single-strain live biotherapeutic product, and this initial data set supports further studies aimed at evaluating ADS024 in future human clinical trials.

## Introduction

The human gut microbiome is composed of a highly diverse network of commensal microorganisms that contribute to health and disease^1–3^. These microorganisms possess an array of chemical capabilities that help modulate host–microbiome interactions in the gut, including vitamin biosynthesis, fermentation of dietary carbohydrates, metabolism of bile, production of indigenous small molecules, and the competitive exclusion of pathogens taking residence in the gut^2,4,5^. The microbiota also influences the development and maturation of the immune system through interactions with the gut epithelium^6^. Through the production of a large variety of compounds, the commensal microbiota contributes to the homeostasis that develops during childhood and remains unique to each person throughout adulthood.

Many factors contribute to the development of a compromised or disrupted microbiome that predisposes the gastrointestinal (GI) tract to colonization with opportunistic pathogens. One such factor is the use of antibiotic therapy to treat routine or suspected bacterial infections^1^. The spectrum of the antibiotics used, length of therapy, and overall exposure influence the degree of disruption to the gut microbiota^7^. Reduced microbial diversity and altered function in the GI tract as a result of antibiotic exposure may contribute to the proliferation of pathogenic bacteria leading to enteric infections such as *Clostridioides* (previously *Clostridium) difficile* infection (CDI) and recurrence of CDI (rCDI)^1,8^.

*C. difficile* infection is a potentially life-threatening illness that has been increasing in prevalence, severity, and mortality^9^. It is the most common healthcare-associated infection in the United States and one of the most common hospital-acquired GI infections around the world^9^. *C*. *difficile* is a spore-forming, opportunistic, anaerobic pathogen that produces up to 3 toxins:—toxin A (TcdA), toxin B (TcdB) or cytolethal distending toxin (CDT)—that are responsible for symptoms ranging from mild diarrhea to more severe pseudomembranous colitis and toxic megacolon^9–11^. Antibiotics for routine or suspected non–*C. difficile* infections are the most widely recognized and modifiable risk factor for initial CDI^7,12^. Other established risk factors include advanced age, hospitalization, and severe comorbid illness^13^. Two major roles for antibiotics in the susceptibility to CDI have been described. First, antibiotics disrupt the barrier function of the normal colonic bacteria, providing an opportunity for *C. difficile* to overgrow and produce TcdA and TcdB^14^. Second, development of *C. difficile* resistance to antibiotics appears to play an important role in disease because of the selection of strains with increased virulence^15,16^.

The broad-spectrum antibiotics recommended for the treatment of CDI reduce GI tract microbial diversity and can further predispose individuals to *C. difficile* overgrowth. Recurrence of CDI, caused by relapse or reinfection, is a serious complication. Recurrent CDI is defined by resolution of CDI symptoms while on appropriate therapy, followed by reappearance of symptoms after initial treatment completion^17^. Up to 25% of patients experience rCDI within 30 days of treatment^18^.

The broad-spectrum antimicrobial activity of standard-of-care antibiotics (including vancomycin and fidaxomicin) used to treat CDI risk of further disruption to the GI microbiota have created a need for alternative strategies for more targeted treatment and prevention of recurrence^19^. Alternative therapies and modalities to prevent recurrence of infection have focused on mitigating disruption to gut microorganisms, restoring the microbiome to promote colonization resistance, limiting colonization by toxigenic *C*. *difficile* strains, and inhibiting the effects of toxins^20–22^.

The only drug approved by the US Food and Drug Administration (FDA) to reduce rCDI is the monoclonal antibody bezlotoxumab, which targets TcdB and is approved for use in adults who are receiving antibacterial treatment for CDI and are at high risk of recurrence^9^. Fecal microbiota transplantation (FMT) is often used in patients with multiple episodes of rCDI to break the cycle of recurrence by replacing their own damaged intestinal microbiome with a donor microbiome, but with varying results^23^. In addition to FMT, emergent therapies under development as live biotherapeutic products (LBPs) for prevention of recurrence include numerous capsule-based standardized microbiota restoration approaches^24–26^. There remains a need for new modalities that can prevent rCDI by directly killing *C. difficile*, inhibiting its toxins and promoting the restoration of a healthy microbiome.

In this study, we report the isolation and initial characterization of ADS024, a *Bacillus velezensis* strain which demonstrated selective killing of *C*. *difficile* and toxin degradation activity. ADS024 is in development as a single-strain LBP (SS-LBP) to prevent rCDI, following a successful treatment with standard-of-care antibiotics, by killing *C. difficile* which remains or germinates from spores, and degrading newly synthesized toxins. In summary, these preclinical analyses suggest that ADS024 is a promising candidate for the prevention of rCDI.

## Results

### Isolation and characterization of ADS024

**Figure 1:**
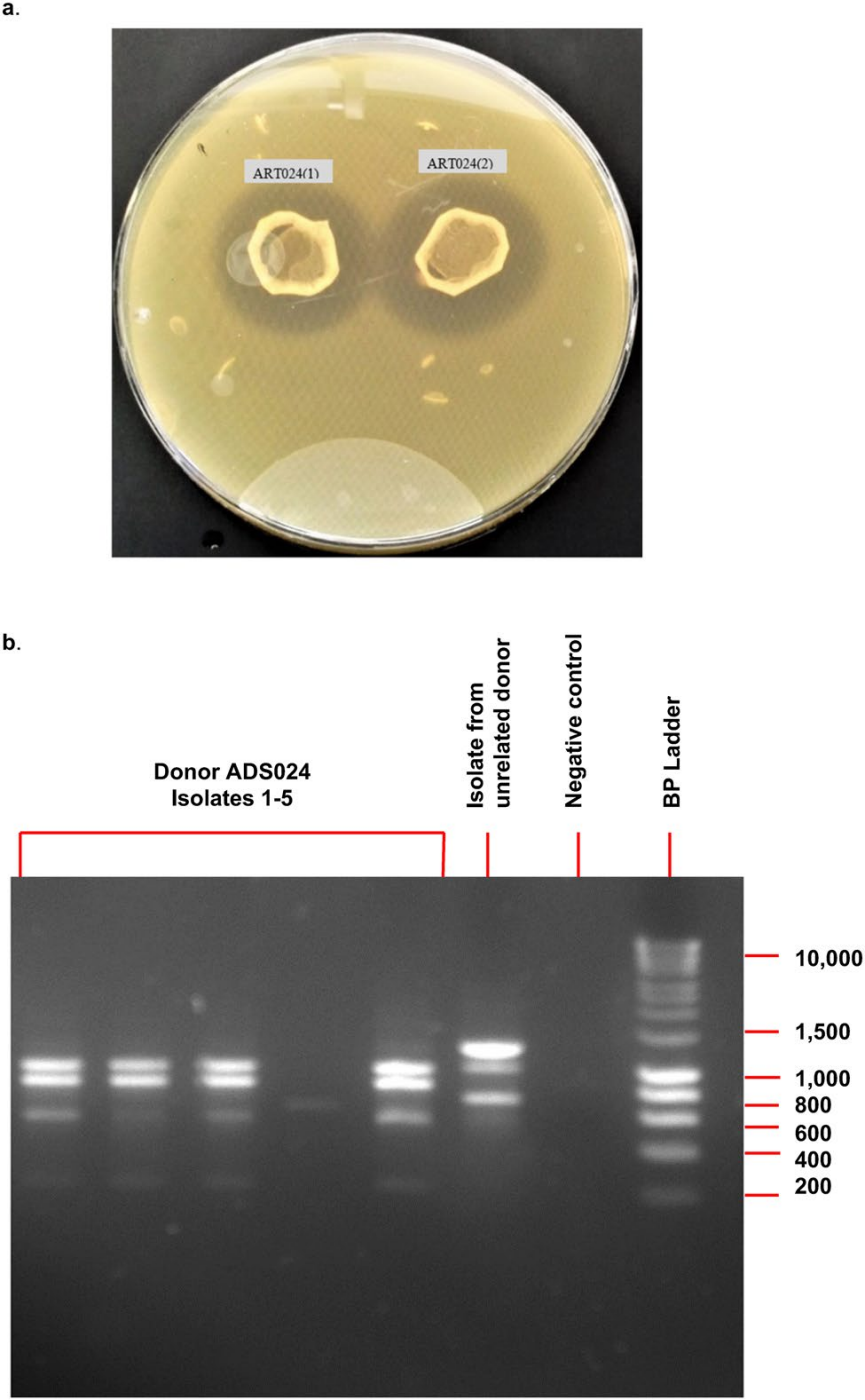
A culture-based screen of fecal samples identified ADS024. (**a**) Representative isolates from a single healthy human donor (ADS024) demonstrating zones of inhibition in a deferred antagonism assay with *C. difficile*. (**b**) Random amplified polymorphic DNA analysis demonstrating that 4 of the 5 isolates belong to the same bacterial strain.

A culture-based screen of aerobic spore-formers resulted in the detection of 5 isolates from a single healthy donor (ADS024), which displayed zones of inhibition against *C. difficile* EM304 in a deferred antagonism assay (Fig. 1a). Random amplified polymorphic DNA (RAPD) profiles suggested that 4 of 5 isolates were of the same profile (Fig. 1b). A single representative was selected for additional characterization and was designated ADS024. ADS024 was selected based on its RAPD banding pattern and potent anti–*C*. *difficile* activity in the deferred antagonism assay. The 16S rRNA sequence of ADS024 was compared to publicly available genes/genomes using nucleotide BLAST (BLASTn). Top hits from BLASTn suggested that ADS024 is a member of the *B.* *amyloliquefaciens*/*B.* *velezensis* operational group.

### ADS024 genome sequencing and speciation

**Figure 2:**
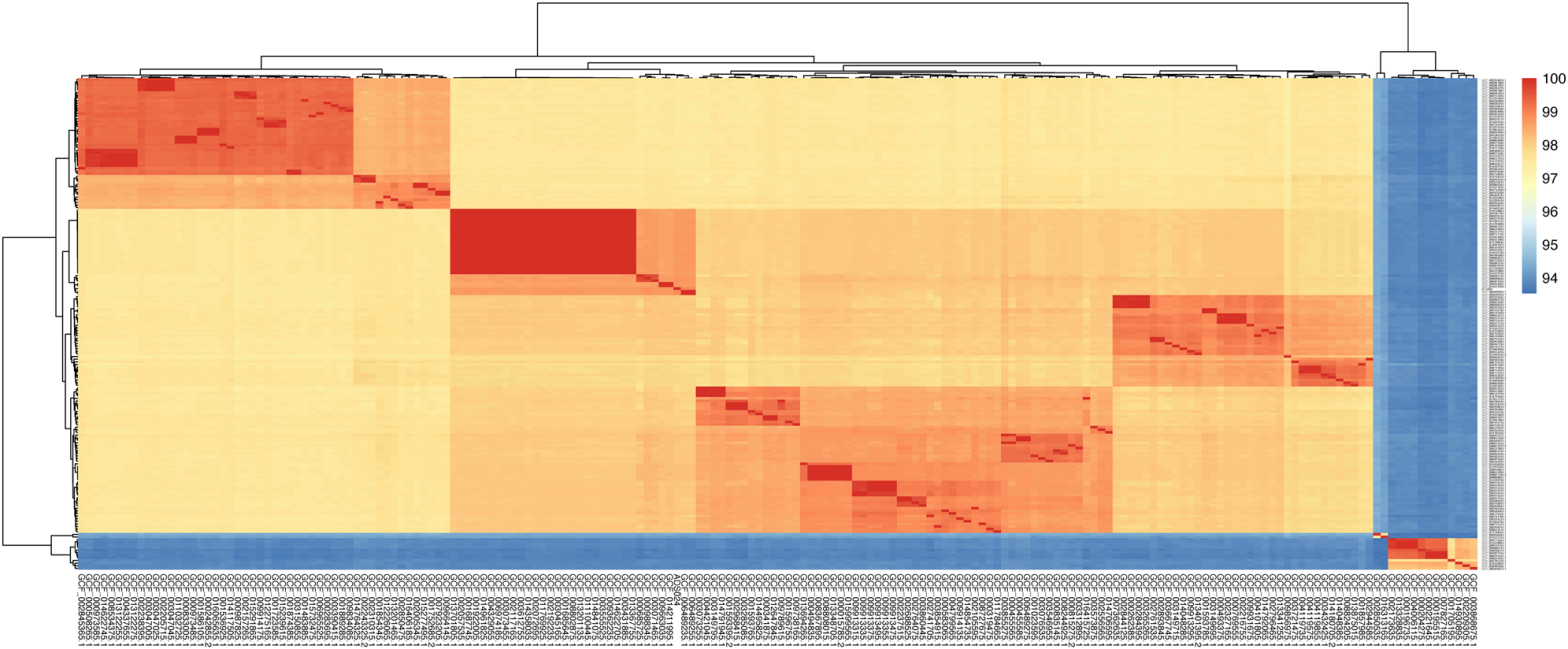
Results of ANI calculation using FastANI for the 187 publicly available genomes of *B. velezensis*, *B. amyloliquefaciens* and *B. siamensis*. Pairwise ANIs were calculated using the tool FastANI (Jain 2018), version 1.1, which computes a very fast and relatively accurate approximation of ANI. FastANI is based on all homologous genome regions between two genomes. Only genome pairs with FastANI exceeding the threshold for species delineation are shown (94%, the hypothetical same-species range).

FastANI was used to compare ADS024 to 187 publicly available genomes of *B. velezensis*, *B. amyloliquefaciens* and *B. siamensis* (Fig. 2). Using this approach ADS024 was more related to *B. velezensis* species. Furthermore, TYGS analysis confirmed ADS024 belongs to the known species *B. velezensis*.

The ADS024 genome was investigated for its antimicrobial gene content, plasmid content, phage content, and virulence factors. A total of 5 prophage regions were identified by PHASTER, which were classified as 2 intact regions and 3 incomplete regions (Supplementary Table 3). No plasmids were identified using the RAST/SEED and IslandViewer 4; no virulence factors or homologs were identified. Approximately 10% of the genome is dedicated to antimicrobial compound production (Tables 1 and 2), with the capacity to produce up to 12 known antimicrobials, including 2 lipopeptides (surfactin and fengycin) and 3 known polyketides produced by *Bacillus subtilis* group bacteria (bacillaene, difficidin, and macrolactin). Other known or associated *B. subtilis* group antimicrobials include the siderophore bacillibactin and the non-thiotemplate, non-ribosomal peptide bacilysin. BAGEL4 analysis revealed 4 areas of interest with a match to known ribosomally synthesized and post-translationally modified peptides or genes encoding bacteriocins.

**Table 1:** ADS024 anti-SMASH results.

Region	Type	From	To	Most similar known cluster		Similarity	MIBiG BGC-ID
Region 1	lanthipeptide	175,852	198,461	Locillomycin	NRPS-t1PKS	35%	BGC0001005
Region 2	NRPS	297,520	361,852	Surfactin	NRPS	86%	BGC0000433
Region 3	PKS-like	915,679	956,923	Butirosin	Saccharide	7%	BGC0000693
Region 4	Terpene	1,041,860	1,061,552				
Region 5	TransAT-PKS	1,342,457	1,432,015	Macrolactin	Other	100%	BGC0000181
Region 6	TransAT-PKS, NRPS	1,657,107	1,757,577	Bacillaene	Other	100%	BGC0001089
Region 7^a^	NRPS, transAT-PKS, betalactone	1,827,578	1,970,090	Fengycin	Other, NRPS	100%	BGC0001095
Region 8	Terpene	1,993,356	2,015,239				
Region 9	T3PKS	2,083,044	2,124,144				
Region 10	TransAT-PKS	2,296,292	2,389,469	Difficidin	Other	100%	BGC0000176
Region 11	NRPS, bacteriocin	3,054,394	3,106,186	Bacillibactin	NRPS	100%	BGC0000309
Region 12	Other	3,626,396	3,667,814	Bacilysin	Other	100%	BGC0001184

^a^Region 7 also identified plipastatin, bacillomycin D, mycosubtilin, iturin and paenilarvin.

*BGC-ID* Biosynthetic gene cluster ID, *MIBIG* Minimum information about a biosynthetic gene, *NPRS* non-ribosomal peptide synthetase, *PKS* polyketide synthase, *t1PKS* type 1 polyketide synthase, *SMASH* Antibiotics and secondary metabolite analysis shell, *transAT PKS* Trans-acyltransferase polyketide synthase.

**Table 2:** Areas of interest (consolidated): results for ADS024 via BAGEL4 analysis.

AOI	Start	End	Class
6666666282745.0.AOI_01	3,090,851	3,111,040	266.1;amylocyclicin
6666666282745.0.AOI_02	277,091	297,226	132.2;LCI
6666666282745.0.AOI_03	660,965	681,379	11.3;colicin
6666666282745.0.AOI_04	177,509	197,509	Lanthipeptide class IV

*AOI* area of interest, *LCI* low CO_2_ (gene type).

### *B. velezensis* isolates from fermented foods do not inhibit *C. difficile*

**Figure 3:**
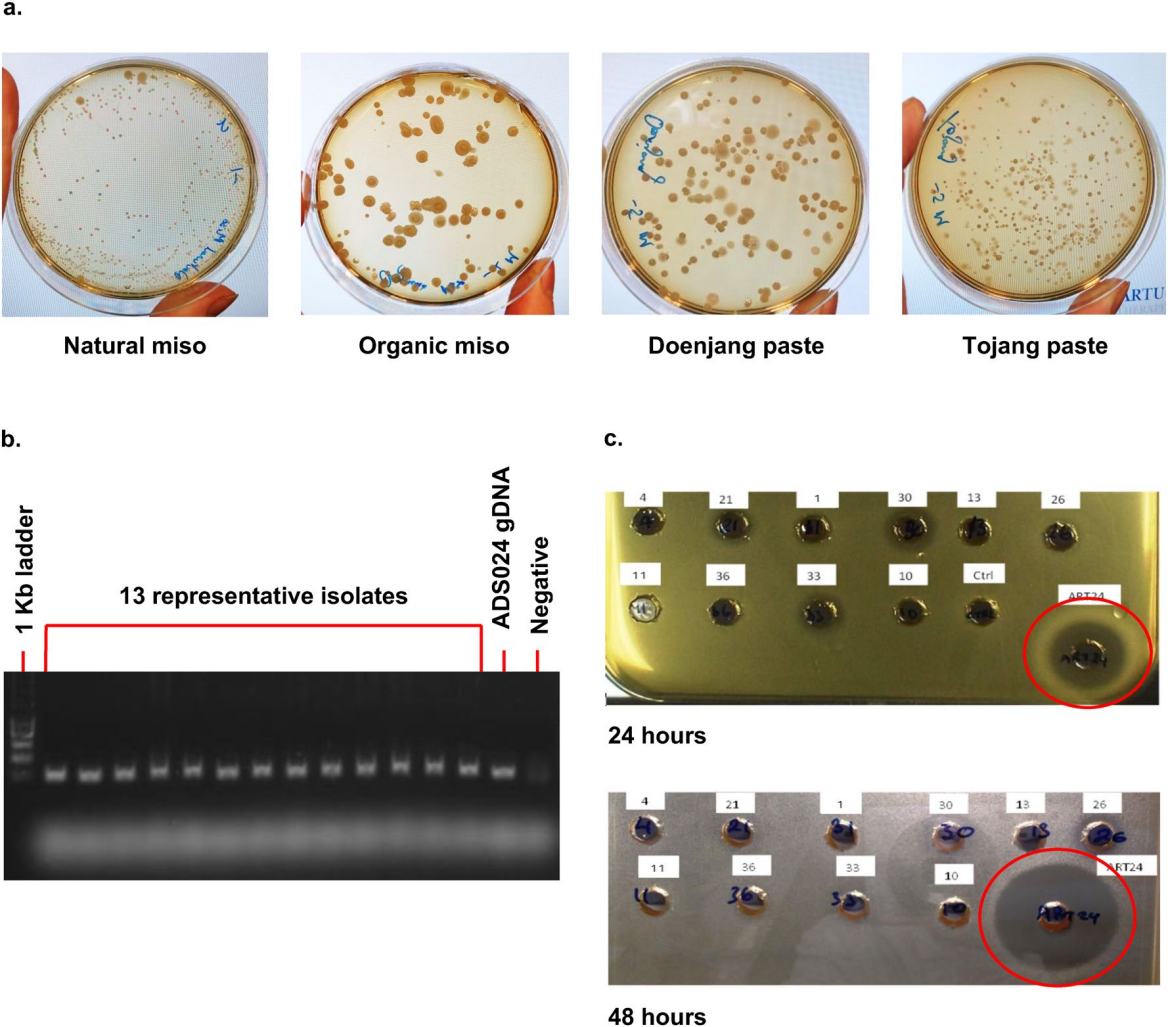
*Bacillus*-like bacteria can be isolated from fermented foods. (**a**) Representative agar plates of cultures of natural miso, organic miso, doenjang paste, and tojang. *B. amyloliquefaciens/B.velezensis* were detected only in organic miso and doenjang paste. (**b**) A subset of 13 representative colony PCR amplicons is shown in (**b**). Colony PCR from 13 representative isolates of *B. amyloliquefaciens/B.velezensis* from fermented foods were compared to ADS024 genomic DNA. Five isolates were selected for whole genome sequencing and confirmed to be *B. velezensis.* (**c**) Supernatants from food-derived *B. amyloliquefaciens* operational group members were compared to supernatant from human-derived ADS024 in the well diffusion assay against *C. difficile.* Only human-derived ADS024 (red circles) displayed clear zones of inhibition after 24 h and 48 h.

It has been reported that fermented foods are a source of *B. velezensis*^27^. Five different fermented foods (natural miso, organic brown rice miso, doenjang soybean paste, tojang paste, and soy sauce) were evaluated for the presence of *B*. *velezensis*. Only organic brown rice miso and doenjang soybean paste contained *Bacillus*-like colonies consistent with *B*. *velezensis* morphology (Fig. 3a). Forty colonies (20 from miso and 20 from doenjang) were selected and used for colony PCR and confirmed to be members of the *B. amyloliquefaciens* operational group. Identical PCR amplicons were amplified from all 40 food isolates (Fig. 3b). Ten isolates with a RAPD PCR banding pattern similar to that of ADS024 were tested for anti-*C. difficile* activity; five of these were sent for WGS. Unlike ADS024, cell-free supernatant (CFS) from *B*. *velezensis* isolated from fermented foods did not inhibit *C*. *difficile* in a well diffusion assay. Isolates sent for WGS were confirmed to be *B. velezensis* (Fig. 3c).

### Anti–*C*.* difficile* activity

**Figure 4:**
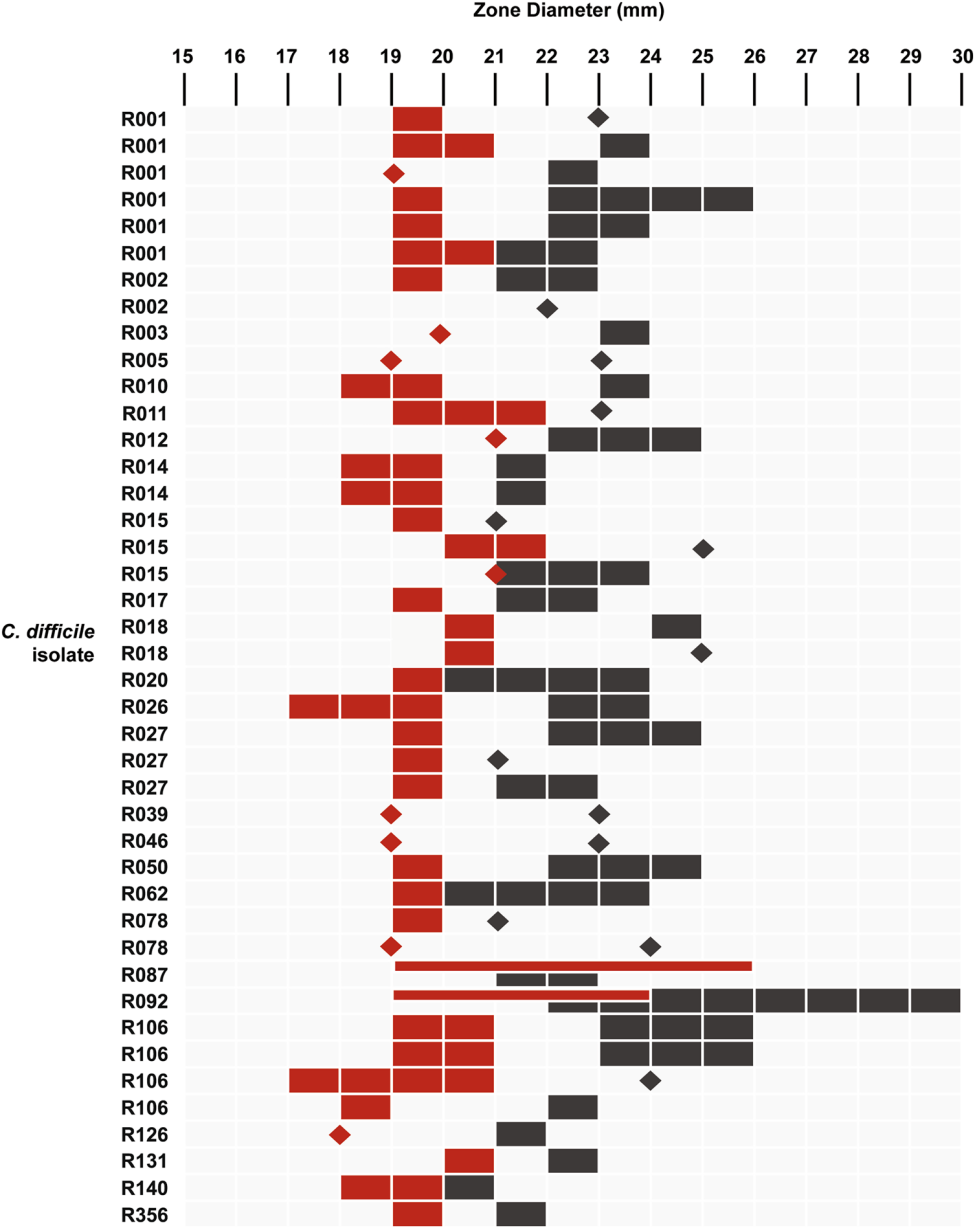
CFS and IPA extracts from ADS024 can inhibit *C. difficile* in a well diffusion assay. Shown are the inhibition zone diameters across 42 *C. difficile* isolates. Diamonds indicate a fixed zone diameter, and rectangles describe a range from two independent experiments. Red indicates CFS; grey indicates IPA extract.

**Figure 5:**
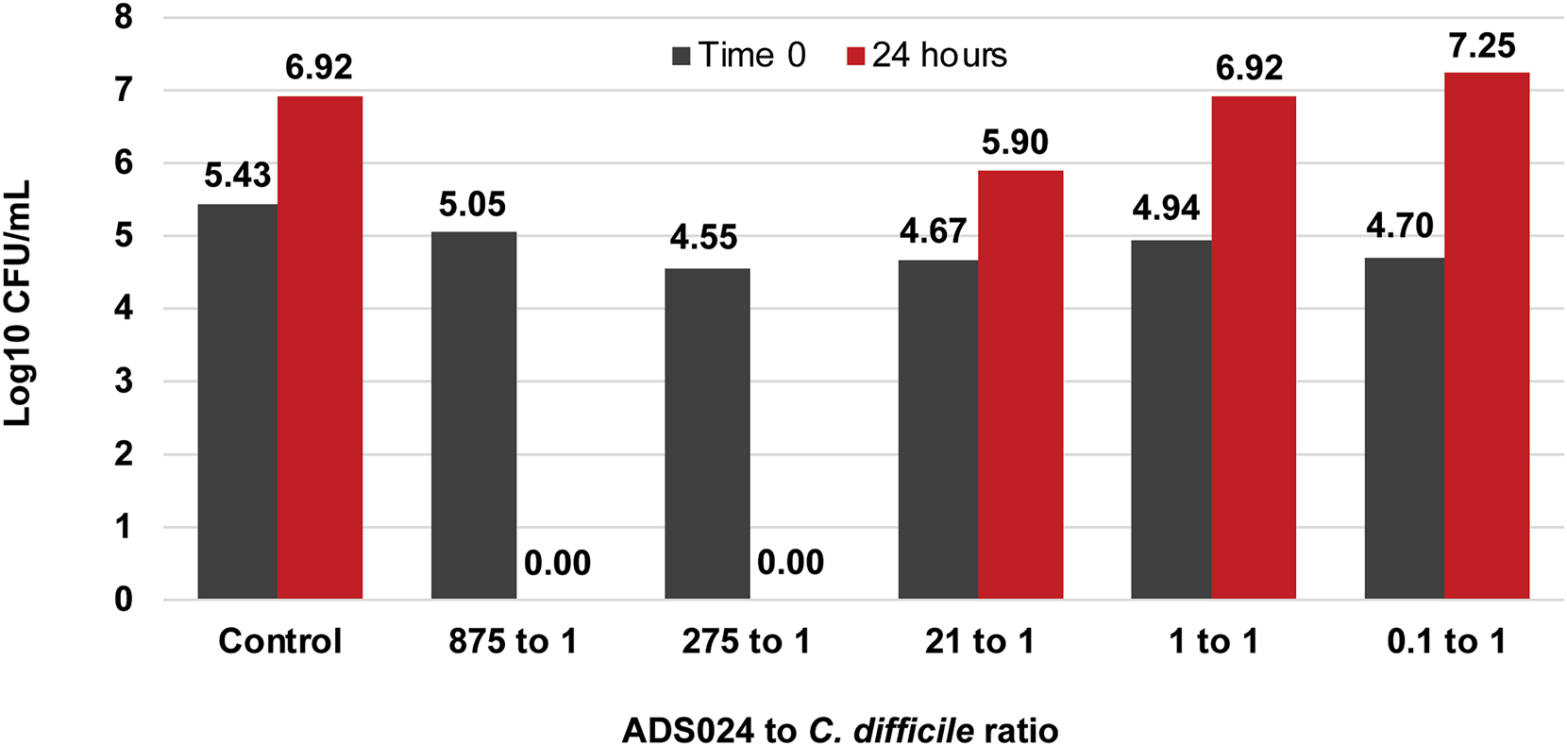
ADS024 can kill *C. difficile* in liquid co-culture. ADS024 was cultured with *C. difficile* at the ratios shown, and *C. difficile* viability was assayed at time zero and 24 h later. Shown is one representative experiment.

ADS024 inhibited all 42 isolates of *C*. *difficile* tested in a well diffusion assay using both isopropanol (IPA) extracts from lyophilized ADS024 cells and CFS from vegetative cells (Fig. 4) (Supp. Table 1). In vitro co-culture experiments further demonstrated the ability of ADS024 to kill *C*. *difficile*. In multiple independent experiments, the lowest ratio of ADS024:*C. difficile* colony-forming units (CFU) that resulted in *C*. *difficile* killing (> 3 log reduction in 24 h) was 275:1; lower ratios inhibited the growth rate of *C*. *difficile* without complete killing (Fig. 5).

### ADS024 degrades *C*. *difficile* toxins A and B

**Figure 6:**
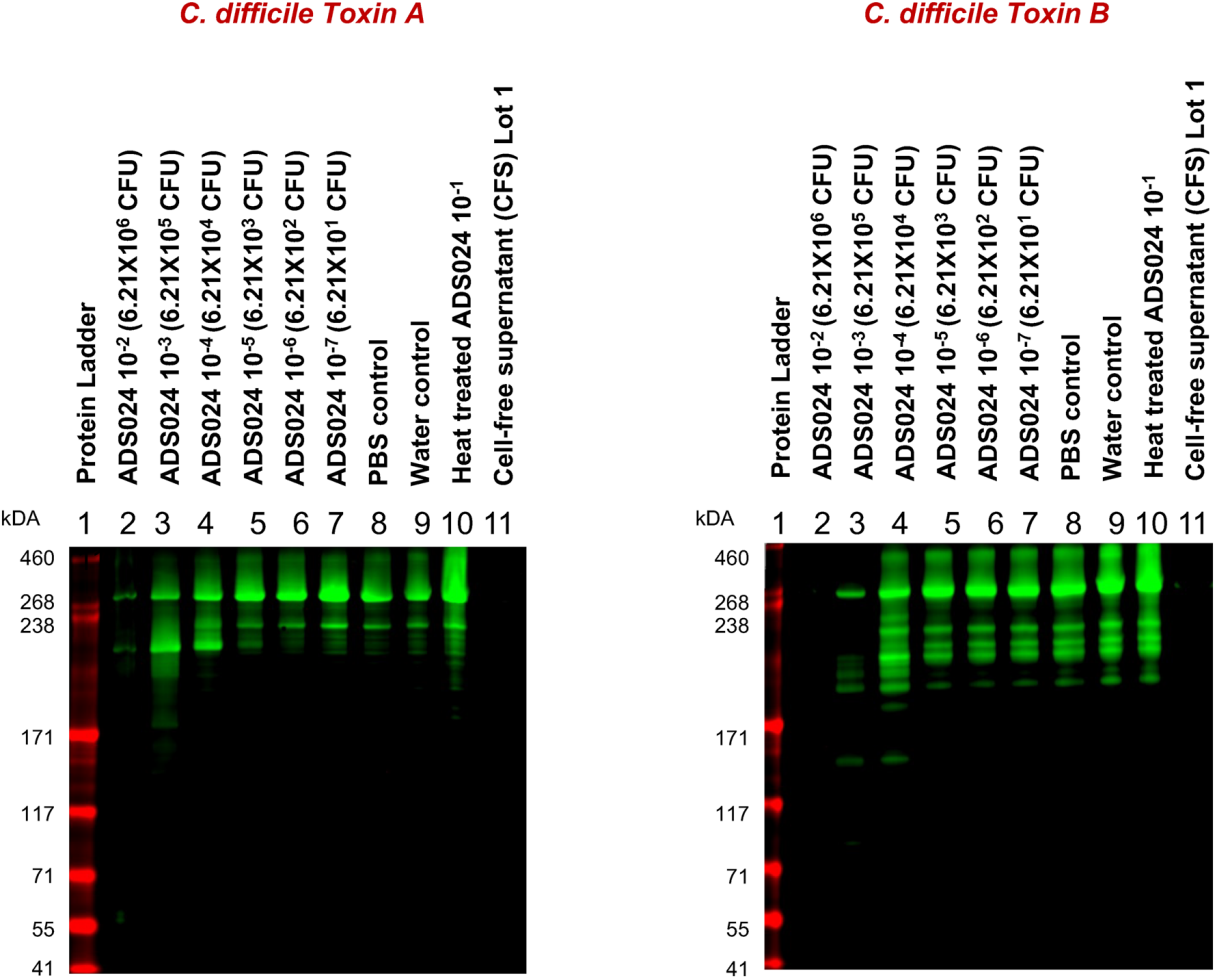
ADS024 CFS, and lyophilate degrade *C. difficile* toxin A and toxin B. Lane 1 shows a Novex Hi Mark pre-stained protein ladder. Toxin A (left) and toxin B (right) were incubated with reconstituted lyophilized ADS024 (lanes 2–7), or CFS (lane 11) in the amounts shown, and toxin proteolytic cleavage was detected by western blot to toxin A and toxin B, respectively. Reconstituted lyophilate was tested once using duplicate samples. Lane 12 and lane 13 use a CFS of Lot 2 and Lot 3, respectively. Images were captured with a Licor Odyssey gel imager. See full uncropped image of Lanes 1–13 in Supplementary Fig. 1 in the supplemental information.

Toxins A and B are important drivers of inflammation and symptoms in patients infected with *C. difficile*. To determine whether ADS024 has the capability to degrade *C. difficile* toxins, both TcdA and TcdB were incubated with ADS024 CFS and reconstituted lyophilized ADS024, and a western blot was performed to detect proteolytic cleavage. The western blot images were made using a Licor Odyssey gel imager. As shown in Fig. 6, CFS caused proteolytic cleavage of *C. difficile* toxins. The same result was observed with reconstituted lyophilized ADS024, with higher amounts of lyophilized ADS024 yielding greater proteolytic cleavage versus lower amounts. ADS024, therefore, possesses the proteolytic capacity to degrade *C. difficile* toxins.

### ADS024 killing activity is limited to *Clostridium* and *Bacillus* species

**Figure 7:**
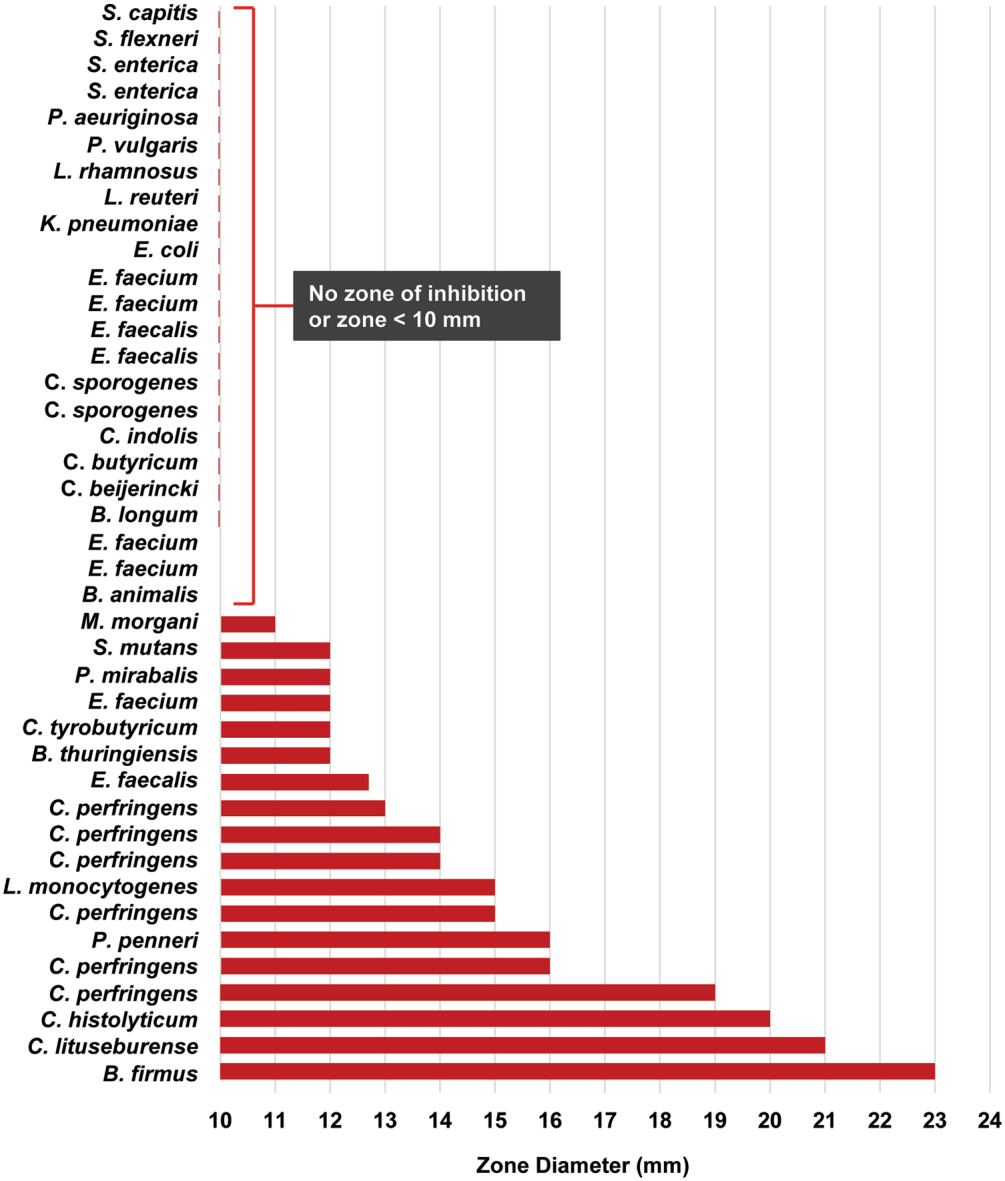
The killing capability of ADS024 is limited to *Clostridium* and *Bacillus* species. The killing activity of ADS024 was evaluated across a panel of gut commensals in a single well diffusion assay using ADS024 CFS. Shown are the zone diameters for each bacterium tested. In cases where the zone diameter was < 10 mm, the zone diameter was noted as “ < 10 mm.” The bore well size was 7 mm, therefore a “10 mm zone” indicates 2–3 mm in actual size. In cases where no zone was visible, the result is listed as “no zone.”

The killing activity of ADS024 was examined against a bank of 41 different gut commensals in a well diffusion assay. ADS024 has activity primarily against spore-forming *C*. *difficile* and *Clostridium* and *Bacillus* species, with limited activity against *Lactobacillus*, *Bifidobacterium*, and other commensals tested (Fig. 7 and Supplemental Table 4). We conclude that the killing capability of ADS024 is selective, with limited impact on other bacterial strains tested.

**Figure 8:**
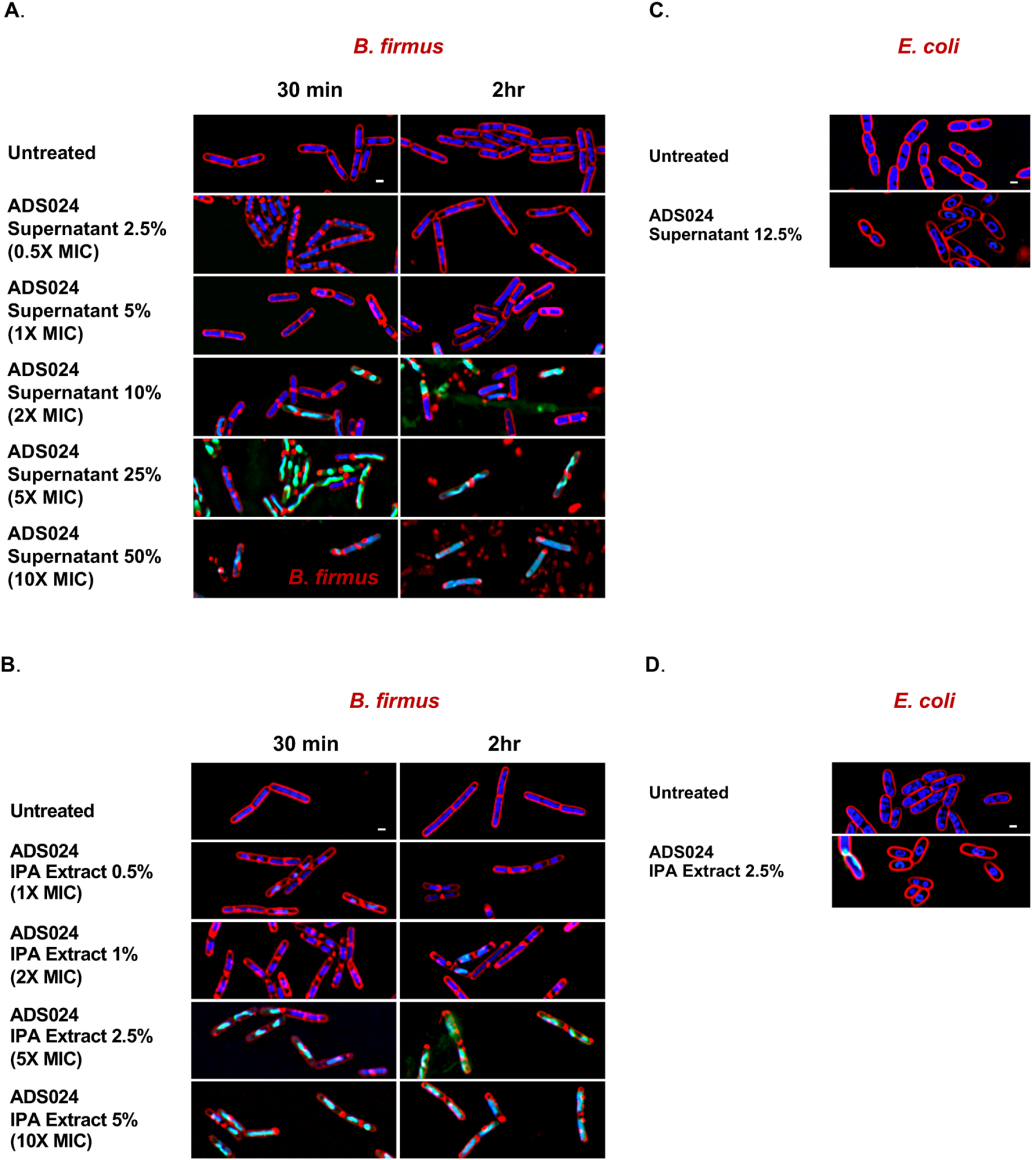
ADS024 CFS and IPA extracts can inhibit translation and disrupt *B. firmus* cell membranes. (**a**) *B. firmus* was incubated with ADS024 CFS for 30 min and 24 h, and bacterial cytological profiling was used to visualize morphological changes. *B. firmus* cell membranes are shown in red, and *B. firmus* DNA is shown in blue. SYTOX Green fluorescence indicates permeabilization of the *B. firmus* cell membrane. (**b**) Similar experiment as in (**a**) except that ADS024 IPA extracts were used. (**c**) Similar experiment as in (**a**) except that *E. coli* was used in place of *B. firmus.* (**d**) Similar experiment as in (**c**) in which *E. coli* was exposed to ADS024 IPA extracts. White scale bar for all images is 1 micron. Experiments were conducted in duplicate.

To better understand the mechanisms by which ADS024 can kill *C. difficile*, bacterial cytological profiling was used to visualize the effect of ADS024 CFS and IPA extracts using a surrogate strain amenable to the experimental conditions. Due to its sensitivity to ADS024, *B. firmus* (Fig. 7) was selected as a surrogate strain and used to give an indication of ADS024’s mechanism of action. Incubation of *B. firmus* with ADS024 CFS and IPA extracts led to disruption and permeabilization of the *B. firmus* cell membrane and inhibition of translation (Fig. 8). In contrast, there was no impact of ADS024 CFS and IPA extracts on *Escherichia coli* by bacterial cytological profiling. Overall, these data suggest that inhibition of translation and permeabilization of the *Bacillus* cell membrane are at least 2 of the potential mechanisms by which ADS024 kills *C. difficile*.

## Discussion

Administration of antibiotics can significantly alter the composition of the gut microbiota, leading to a decreased barrier to pathogen colonization or outgrowth^28^. The use of antibiotics to treat CDI and the impact they have on the gut microbiome underscore the clinical need for treatment modalities that can prevent rCDI. Existing live biotherapeutic products in development represent a promising strategy for the prevention of rCDI following standard-of-care antibiotics, by preventing *C. difficile* colonization through different mechanisms. The aim of this work was to explore whether a SS-LBP’s combined repertoire of metabolites and enzymes offers the potential of simultaneously blocking *C. difficile* growth while also causing inactivation of the pathogen’s toxins.

ADS024 is a strain of *B. velezensis*, a member of the operational group *Bacillus amyloliquefaciens*, which consists of the soil-borne *B. amyloliquefaciens* and the plant-associated *Bacillus siamensis* and *B. velezensis*. Most often, *B. amyloliquefaciens* is used in the literature to describe the operational Group *B. amyloliquefaciens* and refers to all 3 tightly linked species^29^. ADS024 was isolated from a human fecal sample from a healthy donor in Cork, Ireland in 2017. Following a culture-based screen, the most promising isolate was identified as *B*. *velezensis* ^29,30^. Strains of the operational Group *B. amyloliquefaciens* are present in commonly consumed soybean-based foods and have a long history of human consumption ^27,31,32^. However, as our data demonstrate, additional *B*. *velezensis* strains isolated from organic miso and doenjang paste do not possess the *C. difficile–*killing properties of ADS024.

In silico analysis indicated that the ADS024 genome displays all the genetic content expected of *B. velezensis* with low potential for virulence or antibiotic resistance mechanisms. Similar to other *B*. *velezensis* strains, ADS024 dedicates approximately 10% of its genome to the synthesis of antimicrobial compounds. While multiple mechanisms are likely to contribute to the killing activity against *C. difficile*, studies using a surrogate sensitive bacterium suggest that inhibition of translation and destabilization of the bacterial membrane are at least 2 potential contributing mechanisms. In a pan-genome analysis by Chun et al., genes or operons related to the biosynthesis of surfactin, bacillibactin, amylocyclicin, and iturin were identified in all genomes of *B. velezensis* and were also identified in ADS024^33^.

Previous reports have documented that *B. amyloliquefaciens* operational group strains can impede the growth of *C. difficile.* Geeraerts et al. demonstrated antimicrobial activity against all 24 *C*. *difficile* strains tested in a well diffusion assay^34^. Lv et al. also reported the potent inhibition of *C*. *difficile* growth by *B*. *amyloliquefaciens* C-1 lipopeptides surfactin, iturin, and fengycin^35^. The antagonistic activity toward *C*. *difficile* may stem from the ability of certain strains of *B*. *amyloliquefaciens* to produce a number of antimicrobials, including the bacteriocin amylocyclicin, various lipopeptides, and other metabolites that may contribute synergistically to antibacterial activity. Importantly, in this study, ADS024 also inhibits a broad range of clinically relevant *C*. *difficile* strains of different ribotypes in well diffusion assay.

The symptoms of CDI result from the production of toxins, with the amount of toxin produced correlating with the severity of the infection^36^. Toxin B is regarded as the dominant virulence factor^37^. Efforts to neutralize these toxins through proteolytic cleavage have been reported previously for *Bacillus clausii*^38^. We demonstrate that ADS024 CFS and lyophilized whole bacteria can efficiently cleave both TcdA and TcdB in vitro. Future work will examine how this proteolytic function may translate into protection against CDI pathology in in vivo systems. In particular, bacteria belonging to the *Bacillus* genus have been previously demonstrated to have beneficial immunomodulatory effects against intestinal inflammation^39,40^. It remains to be seen whether ADS024 will demonstrate similar properties.

The guideline-recommended antibiotics used to treat CDI disrupt and deplete the gut microbiota, providing an opportunity for CDI to recur following initial antibiotic treatment. Thus, we recognize the importance of finding a mechanism of action that would be predicted to have minimal effect on the gut microbiome. Despite its ability to produce several antimicrobial compounds, the killing activity of ADS024 is most pronounced against *Clostridium* and *Bacillus* species, with limited activity against *Lactobacillus* and *Bifidobacterium* in a well diffusion assay conducted against 41 gut commensals. Future work will seek to extend this observation and to understand the impact on the larger gut microbiome both in vivo and ex vivo.

In summary, our initial results demonstrate ADS024’s dual mechanisms of direct killing activity on *C. difficile* and degradation activity of *C. difficile* toxins. This initial research supports advanced study of ADS024 in human testing and continued investigation as a candidate drug product for the treatment and prevention of CDI.

## Conclusion

The treatment and prevention of rCDI remains a paramount challenge because of the prevalence of CDI and the use of current standard-of-care antibiotics that themselves destabilize natural gut bacteria. In vitro investigations to date suggest that ADS024 represents a novel therapeutic bacterium for the treatment or prevention of rCDI in humans. ADS024 potently inhibits the growth of *C. difficile* and degrades TcdA and TcdB while displaying restricted activity against other bacterial species, suggesting that ADS024 may not interfere with the recovery of the gut microbiome following treatment with standard-of-care antibiotics. The maintenance of the natural bacterial composition within the gut remains a critical factor in the prevention of rCDI and in overall gut health. Additional studies underway will seek to further characterize the ability of ADS024 to protect against CDI in vivo while being microbiome sparing in preparation for first-in-human efficacy and safety studies.

## Materials and methods

### Bacterial strains, media, and culture conditions

All strains isolated from human fecal samples were cultured aerobically at 37 °C for 24 h using brain heart infusion (BHI) agar. *Clostridioides difficile* EM304, a previously reported strain, was selected as the target strain for screening and was cultured anaerobically at 37 °C for 24 h in BHI broth^41^. All other *Clostridioides* strains for use in antimicrobial activity assays and their respective growth conditions are listed in Supplementary Table 1. All other indicator strains used in antimicrobial activity assays and their respective growth conditions are listed in Supplementary Table 2. Anaerobic conditions were maintained throughout using an anaerobic chamber (10% CO_2_, 10% H_2_, 80% N_2_). Where solid media were required, 1.5% agar (w/v) (Oxoid, Basingstoke, UK) or 2.5% agar (w/v) was added for growth of *Bacillus*, and for overlay assays, 0.75% agar (w/v) was added.

### Screening for anti–*C. difficile* activity

Fecal samples were acquired from 20 human donors following ethical approval through the Clinical Research Ethics Committee of the Cork Teaching Hospital^41^. All methods were performed in accordance with the relevant guidelines and regulations^41^. Informed consent was obtained from all subjects to process and use these samples^41^. All volunteers were healthy and had not received antibiotic treatment in the last 6 months. One gram of fecal sample was homogenized with 9 mL maximum recovery diluent (MRD; Oxoid) in a stomacher for 1 min. An equal volume of 70% ethanol was added to the fecal slurry and was allowed to stand at room temperature for 2 h. The slurry was serially diluted tenfold until the 10^–8^ dilution was achieved, and 100 µL of each dilution was plated on BHI agar. Plates were incubated aerobically at 37 °C for 24 h before being examined for CFU. Colonies were overlaid with *C*. *difficile* EM304 using the deferred antagonism assay with 0.75% agar (w/v) and were incubated anaerobically for a further 24 h^42^. Potential anti–*C*. *difficile* isolates were identified by a zone of inhibition surrounding the colony. Colonies displaying zones of inhibition were picked, re-streaked on BHI agar to confirm purity, and grown overnight in BHI broth. Isolates were then stocked in a final concentration of 25% glycerol and stored at  − 80 °C for further characterization.

### Genetic characterization of isolates

Genomic DNA was extracted from isolates using the GenElute Bacterial Genomic DNA Kit (Sigma) as per the manufacturer’s instructions. Randomly amplified polymorphic DNA analysis was performed using the random primer PER1 (5’ AAGAGCCCGT-3’) using only 1 primer in a single PCR reaction^43^. Template DNA (50 ng) and primer (final concentration of 0.4 μM each) were added to a 25 μL reaction mixture of PCR master mix. Amplifications were performed with initial template denaturation at 94 °C for 5 min, followed by 35 cycles of 1 min at 94 °C, an annealing step of 1 min at 36 °C, and extension for 2 min at 72 °C, with the final extension for 10 min at 72 °C. The PCR products obtained were analyzed by agarose gel electrophoresis.

The 16S rRNA sequence of isolates was amplified by PCR using 27F and 1492R primers and was sequenced by Cogenics^44^. A contig (combining the forward and reverse read) was compared to publicly available genes and genomes using BLASTn. Genomic libraries were prepared from the genomic DNA using the Nextera XT library preparation kit (Illumina, San Diego, CA, USA). Whole genome sequencing of ADS024 was performed using the Illumina MiSeq and the Oxford Nanopore MinION platforms at Teagasc Food Research Centre, Fermoy, Co. Cork, Ireland. For Illumina reads quality control, Trimmomatic (v. 0.38) was used to remove adaptors and low-quality sequences and to discard low-quality reads. FastQC (v. 0.11.8) was used to visualize and interpret overall quality. For Nanopore reads quality control, Porechop (v. 0.2.4) was used to remove adaptors and demultiplex from raw FASTQ files. Filtlong (v. 0.2.0) was used to discard any read shorter than 1 kbp, discard the shortest 10% of remaining reads, and discard the reads with lowest Phred quality until 500 Mbp of sequence data remained. Unicycler (v. 0.4.7) was used to perform a hybrid assembly using both Illumina and Nanopore data sets using default parameters and removing any sequences shorter than 500 bp. The assembled genomes were annotated using the RAST tool kit on the web service PATRIC (v. 3.5.39).

FastANI was used to determine the average nucleotide identity between ADS024 and 187 publicly available genomes of *B. velezensis*, *B. amyloliquefaciens*, *B. siamensis* using a recommended cut-off of 94%^45^. The Type (strain) Genome Server was used to perform phylogenomic classification ^46^.

The antimicrobial gene content of ADS024 was analyzed using the antiSMASH 5.0 web server and the BAGEL4 web server. Plasmid content was assessed by plasmidSPAdes, and phage content was assessed by PHASTER. Potential virulence factors of ADS024 were analyzed using IslandViewer 4 and RAST SEED viewer.

### Screening of food products for *B. velezensis*

Five different fermented foods (natural miso, organic brown rice miso, doenjang soybean paste, tojang paste, and soy sauce) were evaluated for the presence of *B*. *velezensis* using culture-based and colony PCR approaches. To screen for the presence of *Bacillus*-like colonies, 1 g of food was placed into a filtered stomacher bag, and 9 mL of MRD was added. The bag was inserted into a stomacher and homogenized for 1 min on a medium setting. The resulting liquid was serially diluted tenfold to achieve 10^–8^ dilution, and 100 µL of each dilution was plated on BHI 2.5% agar. Plates were incubated at 37 °C for 18–24 h before being examined for *Bacillus*-like colony morphologies. Colony PCR was used to further characterize isolates by RAPD analysis and *rpoB* sequencing. Forty isolates (20 organic miso and 20 doenjang) were selected for colony PCR to confirm the presence of operational group *B. amyloliquefaciens* members using primers specific to this group. A further 5 isolates were selected for whole genome sequencing as previously described.

### ADS024 antimicrobial activity assays

The agar well diffusion method was used to assess the antimicrobial activity of the CFS and IPA extract of each isolate. The CFS was prepared from an overnight culture grown shaking in BHI and harvested by centrifuging at 4000 rpm for 20 min. The CFS was adjusted to a pH of 7.0 using 1 M HCl and was filter sterilized using 0.22-μm syringe filters (Sarstedt, Leicester, UK). To solubilize membrane bound metabolites, IPA extracts were prepared by resuspending lyophilized ADS024 in 5 mL IPA and vortexing for 10 min before centrifuging at 4000 rpm for 20 min and filter-sterilizing using 0.22-μm syringe filters. IPA extracted samples were dried using a SpeedVac vacuum and were resuspended in 5 mL BHI. Individual well diffusion assays were performed with 50 µL of CFS or resuspended IPA extracted samples in 6-well plates (Sarstedt). The inhibitory spectrum was assessed against 42 contemporary *C*. *difficile* isolates of 25 distinct ribotypes (Supplementary Table 1). Plates were examined for zones of inhibition following overnight incubation. The spectrum of activity was also examined against a range of gut commensals (Supplementary Table 2) using the well diffusion method.

### Liquid co-culture assay

The direct inhibitory activity of ADS024 was investigated using a 12-well plate liquid co-culture method with *C*. *difficile* VPI10463 (ATCC 43,255). Briefly, an overnight culture of *C. difficile* VPI10463 was diluted in 80 mL BHI until a count of 5 × 10^4^ CFU/mL was achieved, and 3 mL was aliquoted in each well of the plate. Capsules of lyophilized ADS024 were resuspended 1-in-10 in MRD, serially diluted, and plated on BHI agar to determine CFU/mL. A second 1-in-10 dilution series was prepared for use in the co-culture. Dilutions 10^–2^–10^–6^ were centrifuged at 4000 rpm for 10 min, and the supernatant was removed. Cell pellets were resuspended in individual wells containing *C*. *difficile*. Control wells contained *C*. *difficile* only. Counts were enumerated at T0 and T24 to determine *C*. *difficile* numbers by spot plating on CCEY agar (Lab M, Lancashire, UK). The ratio of ADS024 cells to *C*. *difficile* cells was then calculated.

### *Bacillus firmus* mechanism of killing assay

The CFS and IPA extracts were prepared as previously described and used in in vitro killing assays with *B. firmus*, an aerobic indicator strain susceptible to ADS024-mediated killing. Bacterial cytological profiling was used to investigate the effect of ADS024 CFS and IPA extracts on *B. firmus* growth and viability. *B. firmus* was exposed to CFS and IPA extracts for 30 min or 120 min and then stained with 0.4 μM SYTOX Green, 3 μg/mL DAPI, and 1.5 μg/mL FM 4-64 to visualize cell permeability, DNA, and the cell membrane, respectively. Fluorescent microscopy was used to visualize the effects of ADS024 CFS and IPA extracts on *B. firmus* cellular integrity. Repeated experiments were performed with *E. coli* as a negative control.

### Toxin degradation assay

Toxin degradation was assessed using ADS024 CFS and reconstituted lyophilized cells. The CFS was prepared as previously described and was serially diluted 1:2 in BHI broth 10 times. Lyophilized ADS024 was reconstituted and serially diluted in phosphate-buffered saline. Purified *C*. *difficile* toxins A and B (0.2 mg/mL) were obtained from List Biological Laboratories (Campbell, CA, USA). Purified toxin was reconstituted in sterile water to contain 50 mM Tris, 50 mM NaCl, pH 7.5, and 0.1% trehalose and was stored at 2–8 °C; 2.5-μL (0.2 mg/mL) aliquots of purified *C. difficile* toxin A or B were co-incubated for 2 h at 37 °C with 30 μL of either ADS024 CFS, or lyophilized ADS024, and the reactions were stopped, heated, and electrophoresed in a 3–8% NuPAGE Tris–acetate protein gel as 15-μL volumes/well. The gel was electrophoresed at 150 V for 50 min. Western blot using TcdA or TcdB primary antibody (1:5000 dilution from List Biological Laboratories) and fluorescent secondary antibodies was performed.

## Supplementary Information

Supplementary Information 1.

Supplementary Information 2.

Supplementary Information 3.
