# Supplementary material for: Identification of ADS024, a newly characterized strain of Bacillus velezensis with direct Clostridiodes difficile killing and toxin degradation bio-activities
Source: Sci Rep. 2022 Jun 3;12:9283. doi: 10.1038/s41598-022-13248-4 (PMC9166764; doi:10.1038/s41598-022-13248-4)
Supplement: Supplementary file 1 — Supplementary Information 1. [file 41598_2022_13248_MOESM1_ESM.docx]

**Supplementary Figure Legends:**

- Supplementary Figure 1: Cleavage detection of *C. difficile* toxin A and toxin B by reconstituted lyophilized ADS024 by western blot

**Supplementary Table Legends:**

- Supplementary Table 1: *C*. *difficile* isolates used for screening and antimicrobial activity assays
- Supplementary Table 2: Bacterial genera investigated in the well diffusion assay with ADS024
- Supplementary Table 3: Prophage regions of ADS024 genome identified by PHASTER analysis
- Supplementary Table 4: ADS024 activity against commensals and pathobionts

**Supplementary Figure 1 Legend**

**Supplementary Figure 1** shows the full uncropped image, including lanes 1-13, of ADS024 CFS, and reconstituted lyophilate degrade *C. difficile* toxin A and toxin B (Figure 6). Lane 1 shows a Novex Hi Mark pre-stained protein ladder. Toxin B was incubated with reconstituted lyophilized ADS024 (lanes 2-7), or CFS (lanes 11-13) in the amounts shown, and toxin proteolytic cleavage was detected by western blot to toxin B. Reconstituted lyophilate was tested once using duplicate samples. Image was captured with a Licor Odyssey gel imager. Lane 12 and lane 13 use a CFS of Lot 2 and Lot 3. Lanes 12 and 13 were cropped out of Figure 6 in the main manuscript.

**Supplementary Tables**

**Supplementary Table 1**

***C*. *difficile* isolates used for screening and antimicrobial activity assays**

| APC No. | Symptomatic vs. Asymptomatic  carrier | Ribotype | Culture conditions |
| --- | --- | --- | --- |
| **3** | Asymptomatic carrier | R003 | Anaerobic at 37°C, BHI |
| **4** | Asymptomatic carrier | R010 | Anaerobic at 37°C, BHI |
| **5** | Asymptomatic carrier | R020 | Anaerobic at 37°C, BHI |
| **8** | Asymptomatic carrier | R062 | Anaerobic at 37°C, BHI |
| **9** | Asymptomatic carrier | R050 | Anaerobic at 37°C, BHI |
| **11** | Asymptomatic carrier | R026 | Anaerobic at 37°C, BHI |
| **12** | Asymptomatic carrier | R131 | Anaerobic at 37°C, BHI |
| **17** | Asymptomatic carrier | R001 | Anaerobic at 37°C, BHI |
| **18** | Asymptomatic carrier | R106 | Anaerobic at 37°C, BHI |
| **24** | Symptomatic carrier | R001 | Anaerobic at 37°C, BHI |
| **25** | Symptomatic carrier | R018 | Anaerobic at 37°C, BHI |
| **26** | Symptomatic carrier | R018 | Anaerobic at 37°C, BHI |
| **27** | Symptomatic carrier | R106 | Anaerobic at 37°C, BHI |
| **28** | Symptomatic carrier | R106 | Anaerobic at 37°C, BHI |
| **40** | Symptomatic carrier | R001 | Anaerobic at 37°C, BHI |
| **41** | Symptomatic carrier | R001 | Anaerobic at 37°C, BHI |
| **43** | Asymptomatic carrier | R078 | Anaerobic at 37°C, BHI |
| **1202** | Asymptomatic carrier | R017 | Anaerobic at 37°C, BHI |
| **1203** | Asymptomatic carrier | R012 | Anaerobic at 37°C, BHI |
| **1207** | Symptomatic carrier | R001 | Anaerobic at 37°C, BHI |
| **1208** | Symptomatic carrier | R015 | Anaerobic at 37°C, BHI |
| **1209** | Symptomatic carrier | R106 | Anaerobic at 37°C, BHI |
| **1211** | Symptomatic carrier | R027 | Anaerobic at 37°C, BHI |
| **1212** | Symptomatic carrier | R002 | Anaerobic at 37°C, BHI |
| **1213** | Symptomatic carrier | R027 | Anaerobic at 37°C, BHI |
| **1214** | Asymptomatic carrier | R014 | Anaerobic at 37°C, BHI |
| **1398** | Asymptomatic carrier | R014 | Anaerobic at 37°C, BHI |
| **1399** | Asymptomatic carrier | R002 | Anaerobic at 37°C, BHI |
| **1401** | Asymptomatic carrier | R126 | Anaerobic at 37°C, BHI |
| **1404** | Asymptomatic carrier | R140 | Anaerobic at 37°C, BHI |
| **1410** | Asymptomatic carrier | R015 | Anaerobic at 37°C, BHI |
| **1412** | Asymptomatic carrier | R046 | Anaerobic at 37°C, BHI |
| **1418** | Asymptomatic carrier | R039 | Anaerobic at 37°C, BHI |
| **1421** | Asymptomatic carrier | R078 | Anaerobic at 37°C, BHI |
| **1424** | Asymptomatic carrier | R011 | Anaerobic at 37°C, BHI |
| **1425** | Asymptomatic carrier | R005 | Anaerobic at 37°C, BHI |
| **1427** | Asymptomatic carrier | R092 | Anaerobic at 37°C, BHI |
| **1431** | Asymptomatic carrier | R087 | Anaerobic at 37°C, BHI |
| **1432** | Asymptomatic carrier | R356 | Anaerobic at 37°C, BHI |
| **1441** | Asymptomatic carrier | R001 | Anaerobic at 37°C, BHI |
| **1442** | Asymptomatic carrier | R015 | Anaerobic at 37°C, BHI |
| **1451** | Asymptomatic carrier | R027 | Anaerobic at 37°C, BHI |

APC = APC Microbiome Ireland; BHI = Brain heart infusion

Supplementary Table 2

Bacterial genera investigated in the well diffusion assay with ADS024

| **Genus** | **Growth medium** | **Atmospheric Conditions** |
| --- | --- | --- |
| *Bacillus* | BHI | Aerobic |
| *Bifidobacterium* | mMRS | Anaerobic |
| *Clostridium* | RCA | Anaerobic |
| *Enterococcus* | MRS | Aerobic |
| *Escherichia* | LB | Aerobic |
| *Klebsiella* | Nutrient agar | Aerobic |
| *Lactobacillus* | MRS | Anaerobic |
| *Listeria* | BHI | Aerobic |
| *Morganella* | Nutrient agar | Aerobic |
| *Proteus* | Nutrient agar | Aerobic |
| *Pseudomonas* | LB | Aerobic |
| *Salmonella* | BHI | Aerobic |
| *Shigella* | LB | Aerobic |
| *Staphylococcus* | BHI | Aerobic |
| *Streptococcus* | BHI | Aerobic |

BHI: Brain heart infusion, LB: Luria-Bertani, RCA: Reinforced clostridial agar, MRS: de Man, Rogosa and Sharpe agar, mMRS: modified de Man, Rogosa and Sharpe agar

**Supplementary Table 3
Prophage regions of ADS024 genome identified by PHASTER analysis**

| **Region** | **Region Length** | **Complete-ness** | **Score** | **# Total Proteins** | **Region Position** | **Most Common Phage** | **GC %** |
| --- | --- | --- | --- | --- | --- | --- | --- |
| 1 | 13.2Kb | Incomplete | 10 | 27 | [1184349-1197612 *info_outline*](http://phaster.ca/submissions/ZZ_9448a3468e#region_dna0) | PHAGE_Bacill_SPbeta_NC_001884(7) | 46.01% |
| 2 | 26.5Kb | Incomplete | 50 | 12 | [1782484-1809025 *info_outline*](http://phaster.ca/submissions/ZZ_9448a3468e#region_dna1) | PHAGE_Bacill_AR9_NC_031039(4) | 41.07% |
| 3 | 39.8Kb | Intact | 110 | 60 | [2126479-2166377 *info_outline*](http://phaster.ca/submissions/ZZ_9448a3468e#region_dna2) | PHAGE_Deep_s_D6E_NC_019544(12) | 43.08% |
| 4 | 54.7Kb | Intact | 120 | 48 | [2649118-2703850 *info_outline*](http://phaster.ca/submissions/ZZ_9448a3468e#region_dna3) | PHAGE_Bacill_phi105_NC_004167(41) | 43.59% |
| 5 | 18.7Kb | Incomplete | 20 | 11 | [3945914-3964683 *info_outline*](http://phaster.ca/submissions/ZZ_9448a3468e#region_dna4) | PHAGE_Bacill_SPbeta_NC_001884(4) | 42.17% |

Supplementary Table 4

ADS024 activity against commensals

| Commensals | | **Zone diameter (mm)** |
| --- | --- | --- |
| Genus | Species |  |
| *Bacillus* | *firmus* | 23 |
| *Bacillus* | *thuringiensis* | 12 |
| *Bifidobacterium* | *animalis* | 10 |
| *Bifidobacterium* | *longum* | 8 |
| *Enterococcus* | *faecalis* | 12.7 |
| *Enterococcus* | *faecalis* | NZ |
| *Enterococcus* | *faecalis* | NZ |
| *Enterococcus* | *faecium* | 12 |
| *Enterococcus* | *faecium* | 10 |
| *Enterococcus* | *faecium* | 10 |
| *Enterococcus* | *faecium* | 0 |
| *Enterococcus* | *faecium* | 9 |
| *Escherichia* | *coli* | NZ |
| *Klebsiella* | *pneumoniae* | NZ |
| *Lactobacillus* | *reuteri* | 9.5 |
| *Lactobacillus* | *rhamnosus* | 10 |
| *Listeria* | *monocytogenes* | 15 |
| *Morganella* | *morganii* | 11 |
| *Proteus* | *mirabilus* | 12 |
| *Proteus* | *penneri* | 15.5 |
| *Proteus* | *vulgaris* | 10 |
| *Pseudomonas* | *aeruginosa* | NZ |
| *Salmonella* | *enterica* | NZ |
| *Salmonella* | *enterica* | NZ |
| *Shigella* | *flexneri* | NZ |
| *Staphylococcus* | *capitis* | NZ |
| *Streptococcus* | *mutans* | 12 |

NZ= No zone
