# Supplementary figures and images for: Identification of ADS024, a newly characterized strain of Bacillus velezensis with direct Clostridiodes difficile killing and toxin degradation bio-activities
Source: Sci Rep. 2022 Jun 3;12:9283. doi: 10.1038/s41598-022-13248-4 (PMC9166764; doi:10.1038/s41598-022-13248-4)

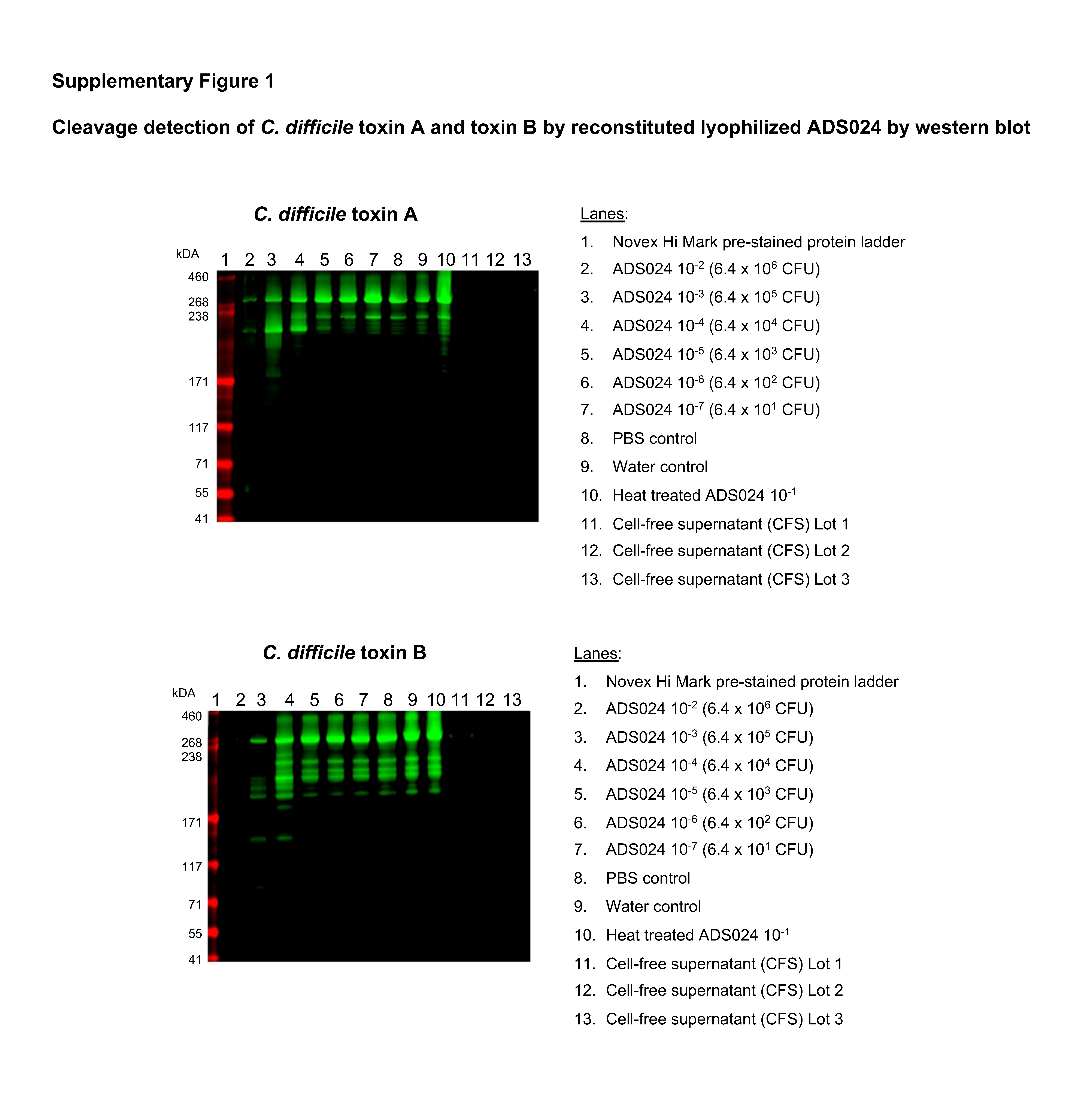

Supplement: Supplementary file 3 — Supplementary Information 3. [file 41598_2022_13248_MOESM3_ESM.tif]
